# Identification of a promoter region specifically active in the maturing endosperm of Arabidopsis seeds and its use for targeted modification of fatty acid metabolism

**DOI:** 10.1111/tpj.70038

**Published:** 2025-03-03

**Authors:** Romane Miray, Sami Kazaz, Alexandra To, Sébastien Baud

**Affiliations:** ^1^ Université Paris‐Saclay, INRAE, AgroParisTech, Institute Jean‐Pierre Bourgin for Plant Sciences (IJPB) 78000 Versailles France

**Keywords:** endosperm, seed, Arabidopsis, maturation, fatty acid, desaturase, promoter, technical advance

## Abstract

In angiosperm seeds, the relative proportions of the two zygotic tissues vary considerably from species to species. In many field‐grown oilseed species, and in those of the model species *Arabidopsis thaliana*, the embryo predominates, and studies of lipid metabolism in whole seeds reflect embryonic metabolism. Metabolism in the endosperm has long been ignored in these species, where this tissue is reduced in size in the mature seed. As a result of recent methodological developments that allow us to follow up on the accumulation of transcripts and metabolites in different areas of these seeds, it has become clear that the lipid metabolism of the endosperm is often different from that of the embryo. However, as the differences between the two zygotic tissues are variations on the same theme rather than strict divergences, there is a lack of genetic tools to study either tissue specifically. To remedy this, we have identified and characterized a promoter sequence in *A. thaliana* that is specifically active in the seed endosperm during the maturation phase: the At3g29190 (*TPS15*) gene promoter. We have then shown that it is possible to use this promoter sequence to modulate fatty acid metabolism specifically in the endosperm, either by activating or repressing the expression of target genes in this tissue. This tool and the transgenic lines that can be generated will contribute to a better understanding of the specific features of lipid metabolism in oilseed endosperm and its physiological implications for the seed.

## Significance Statement

The study of endosperm metabolism and its role in seed physiology requires the ability to specifically modulate it. The At3g29190 (*TPS15*) gene promoter is specifically active in the endosperm of maturing Arabidopsis seeds, and we show that it can be used to specifically alter fatty acid metabolism in this tissue without altering that of the embryo.

## INTRODUCTION

In angiosperms, seeds develop from the fertilized ovule. Double fertilization of the embryo sac initiates the development of two zygotic tissues, the embryo and the endosperm, which grow embedded in maternal tissues composing the seed coat (Butel & Köhler, 2024). The diploid embryo originates from the fertilized egg cell, while the triploid endosperm originates from the fertilized central cell. From an evolutionary perspective, the origin of the endosperm is highly debated. A solution to the century‐old question of its potential homology with an embryo or a female gametophyte remains elusive (Friedman, 1995; Geeta, 2003; Ingram, 2010). The relative contribution of the endosperm to the final mass of the mature seed varies greatly between species (Baroux et al., 2002; Doll & Nowack, 2024; Joët et al., 2009). In Podostemaceae, the endosperm is absent (Khanduri et al., 2016). In the seeds of many dicot species, the endosperm is gradually depleted as the embryo grows during early seed maturation, leaving only one or a few peripheral layers of living cells surrounding a massive embryo, as in Brassicaceae and Fabaceae species (Barthole et al., 2014; Shih et al., 2019). In cereals, the endosperm makes up most of the seed mass in the caryopsis and consists of abundant dead storage tissue, called the starchy endosperm, surrounded by the living aleurone layer (Barthole et al., 2012; Olsen, 2020).

Storage compounds accumulate in the two zygotic tissues during seed maturation. The major storage compounds usually consist of storage proteins, starch or ß‐glucans, and storage lipids such as waxes or triacylglycerols. The relative proportions and locations of these compounds in seeds vary greatly between plant species. Triacylglycerol storage in angiosperm seeds is common, both in seeds with a monolayer aleurone‐like endosperm, such as *Arabidopsis thaliana* or *Brassica napus* (rapeseed), and in seeds with an enlarged endosperm, such as *Ricinus communis* (castor bean) or *Elaeis guineensis* (oil palm) (Baud & Lepiniec, 2010; Dussert et al., 2013; Sturtevant et al., 2019). Triacylglycerols are triesters of fatty acids and glycerol. Fatty acid biosynthesis uses end products of the glycolysis and takes place in the plastids (Troncoso‐Ponce, Nikovics, et al., 2016). In brief, the pyruvate dehydrogenase complex produces acetyl‐coenzyme A (CoA), the building block used for fatty acid synthesis. Biosynthesis begins with the formation of malonyl‐CoA from acetyl‐CoA by acetyl‐CoA carboxylase. The malonyl group of malonyl‐CoA is then transferred to an acyl carrier protein (ACP) by a malonyl‐CoA:ACP malonyltransferase. Saturated acyl chains are produced by the fatty acid synthase complex, which uses acetyl‐CoA as the starting unit, while malonyl‐ACP provides the two‐carbon units required for chain elongation. Some saturated acyl‐ACP can be desaturated by soluble acyl‐ACP desaturases to yield monounsaturated acyl‐ACP (Kazaz et al., 2022). The acyl chains are finally hydrolyzed by acyl‐ACP thioesterases, allowing fatty acids to be exported from the plastid. Fatty acid modification can then continue in many different ways, depending on the plant species (Baud, 2018; Scott et al., 2022). The production of polyunsaturated fatty acids from monounsaturated fatty acids is very widespread and involves FAD‐type membrane desaturases. The production of very long‐chain fatty acids (C > 18) results from the action of the elongase complex. The range of fatty acids thus expanded can ultimately be used to produce triacylglycerol molecules via interconnected and partly redundant metabolic pathways, notably involving the Kennedy pathway and the Lands cycle (Bates, 2016).

Several decades of research have elucidated more than 450 different fatty acid structures and seed fatty acid composition data for over 9000 plants (Aitzetmüller et al., 2003; Ohlrogge et al., 2018), as angiosperms collectively show a huge variation in the fatty acids they store in seeds. Over the last 20 years, the emergence and widespread use of genomic resources and functional genomics tools have enabled the biosynthetic pathways associated with the most common fatty acids to be deciphered, particularly in species of agronomic interest, before the biosynthesis of several unusual fatty acids began to be understood. In the vast majority of cases, these studies have been carried out on whole seeds. Only recently have authors begun to independently analyze and compare lipid metabolism in the two zygotic tissues of the seed (Miray et al., 2021). These approaches have mainly been made possible, particularly in species that produce small seeds, by the continuous improvement of analytical techniques and the ever‐decreasing amounts of material required for RNA or lipid analysis. The combination of laser microdissection techniques and transcriptomic analyses has also proved to be a great help in these comparative analyses (Belmonte et al., 2013; Le et al., 2010), while the recent development of mass spectrometric imaging instruments, including matrix‐assisted laser desorption/ionization mass spectrometry (MALDI‐MS), desorption electrospray ionization mass spectrometry (DESI‐MS) and secondary ion mass spectrometry (SIMS), has bridged the limitations of conventional microscopy and lipid extract analysis, allowing comprehensive metabolite detection *in situ* (Lu et al., 2018). These comparative analyses have shown that the lipid metabolism of the embryo and the endosperm often diverge within the same seed, and that these divergences can even lead to the endosperm becoming almost specialized in the biosynthesis of certain fatty acids. Examples include the endosperm of some Brassicaceae, which accumulate large quantities of omega‐7 monounsaturated fatty acids (Bryant et al., 2016; Penfield et al., 2004), or that of the oil palm, which is enriched in medium‐chain fatty acids (8:0 to 14:0) accounting for roughly 75% of total fatty acids (Dussert et al., 2013).

The differential regulation of lipid metabolism in zygotic seed tissues requires a better understanding of the molecular mechanisms underlying these variations. One example is the transcriptional activation of the genes encoding the palmitoyl‐ACP desaturases AAD2 and AAD3 responsible for omega‐7 fatty acid biosynthesis in the endosperm of *A. thaliana* seeds by the transcription factors MYB115 and MYB118 (Troncoso‐Ponce, Barthole, et al., 2016). On the other hand, these variations also invite us to reflect on the physiological consequences of this differentiated metabolism between zygotic tissues and to question the possible adaptive advantages that it may confer on the species. In this area, it must be said that our knowledge is practically nonexistent. One of the reasons for this is the lack of functional genomics tools to study the specific functions of fatty acids in one seed compartment or another. The available mutants display disrupted metabolic fluxes in all plant tissues in an undifferentiated manner. As for the strategies used so far to modify in a seed‐specific manner the expression of genes involved in lipid metabolism in oilseeds (Belide et al., 2012; Eccleston & Ohlrogge, 1998; Rezzonico et al., 2004; Tian et al., 2018), they usually use promoter sequences of genes encoding reserve proteins (e.g. napins) that are active in both zygotic tissues of the seed (Belmonte et al., 2013; Ettaki et al., 2018; Lorenz et al., 2014; Penfield et al., 2006).

The aim of this study was to identify and validate a promoter sequence specific to the seed endosperm of *A. thaliana*, the activity of which would effectively modulate fatty acid metabolism in this particular seed compartment during the maturation process. Using open‐access transcriptomic data, we identified a gene whose expression pattern met all the requirements, and after cloning its promoter upstream of a reporter gene, we were able to show that it is specifically activated in the seed endosperm during maturation. Finally, we propose three examples of targeted modifications of fatty acid metabolism in the endosperm of *A. thaliana*. In the first example, we used the identified promoter to replace that of a gene naturally induced in the endosperm and showed that it was able to induce omega‐7 accumulation in this tissue. In a second example, we used this promoter to drive the expression of a transgene from *Umbellularia californica* encoding a thioesterase to produce medium‐chain fatty acids in the endosperm, fatty acids that are not normally produced in this tissue. In a third example, this promoter was used to repress *FAD2* expression and reduce the production of polyunsaturated fatty acids specifically in the endosperm.

## RESULTS AND DISCUSSION

### Identification of a gene specifically expressed in the endosperm of maturing *A. thaliana* seeds

**Figure 1:**
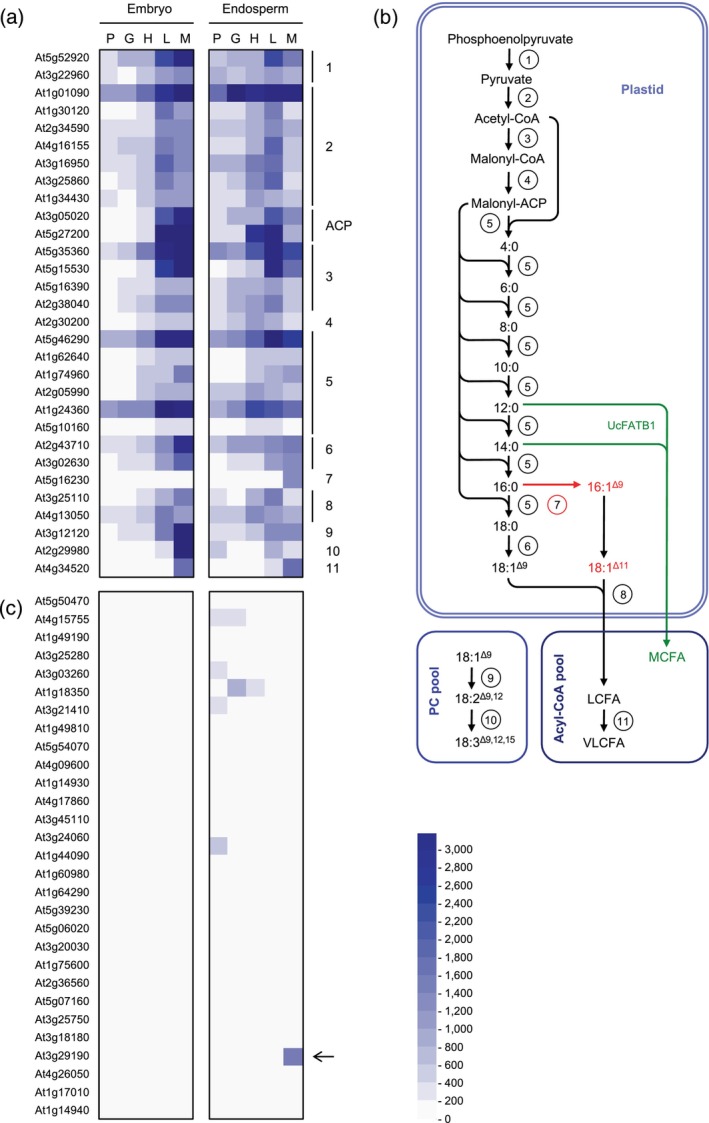
Comparative kinetics of transcript accumulation in endosperm and embryo during *Arabidopsis thaliana* seed maturation. (a) Transcripts encoding enzymes involved in lipid metabolism were examined throughout seed development, from the pre‐globular stage to the maturation green stage, using the ATH1 GeneChip data (Le et al., 2010). Seed parts excised by laser‐capture microdissection and used for RNA extraction that were considered to prepare this figure correspond to the embryo and peripheral endosperm. (b) Simplified schematic representation of fatty acid metabolism in *A. thaliana* seeds. The black arrows represent enzymatic reactions present in the two zygotic tissues of the seed, the red arrows represent enzymatic reactions more specific to the endosperm, and the green arrows represent enzymatic reactions not naturally present in this seed. (c) Endosperm‐specific transcripts were examined in the same tissues as above. 1, pyruvate kinase; 2, pyruvate dehydrogenase; 3, acetyl‐coenzyme A carboxylase; 4, malonyl‐coenzyme A:acyl carrier protein malonyltransferase; 5, fatty acid synthase; 6, stearoyl‐ACP desaturase; 7, palmitoyl‐ACP desaturase; 8, thioesterase; 9, ∆12 fatty acid desaturase; 10, ∆15 fatty acid desaturase; 11, acyl‐CoA elongase; ACP, acyl carrier protein; G, globular stage; H, heart stage; L, linear cotyledon stage; LCFA, long‐chain fatty acid; M, maturation green stage; MCFA, medium‐chain fatty acid; P, pre‐globular stage; PC, phosphatidylcholine; VLCFA, very long‐chain fatty acid.

In the mature *A. thaliana* seed, approximately 90% of the lipid reserves are stored in the massive embryo, which occupies most of the seed space, while the remaining 10% are in the endosperm, a single cell layer located between the embryo and the seed coat (Barthole et al., 2014). Consistent with this dual location of storage lipid biosynthesis, transcriptomic analyses performed on seed parts excised by laser capture microdissection at different stages of seed development (from early embryo morphogenesis to early maturation) allow us to observe the transcriptional activation of the fatty acid metabolic network in the two zygotic tissues at the onset of the maturation process (Belmonte et al., 2013; Le et al., 2010). The abundance of transcripts encoding plastidial enzymes for late glycolysis (pyruvate kinase), biosynthesis of fatty acid biosynthetic precursors (pyruvate dehydrogenase complex, acetyl‐CoA carboxylase, malonyl‐CoA:ACP malonyltransferase), *de novo* fatty acid synthesis (fatty acid synthase complex) and acyl‐ACP hydrolysis (acyl‐ACP thioesterase) shows a marked increase in both zygotic tissues once the embryo has passed the heart stage, 6 days after flowering (DAF), and begins to elongate, initiating the maturation process (Figure 1a,b). A similar trend is observed for the transcripts encoding the plastidial ∆9 steroyl‐ACP desaturases, the endoplasmic reticulum‐localized ∆12 desaturase FAD2 and ∆15 desaturase FAD3. A slightly delayed activation is observed in both seed compartments for the *KETO‐ACYL SYNTHASE 18* (*KCS18*), which encodes a component of the elongase complex.

**Figure 2:**
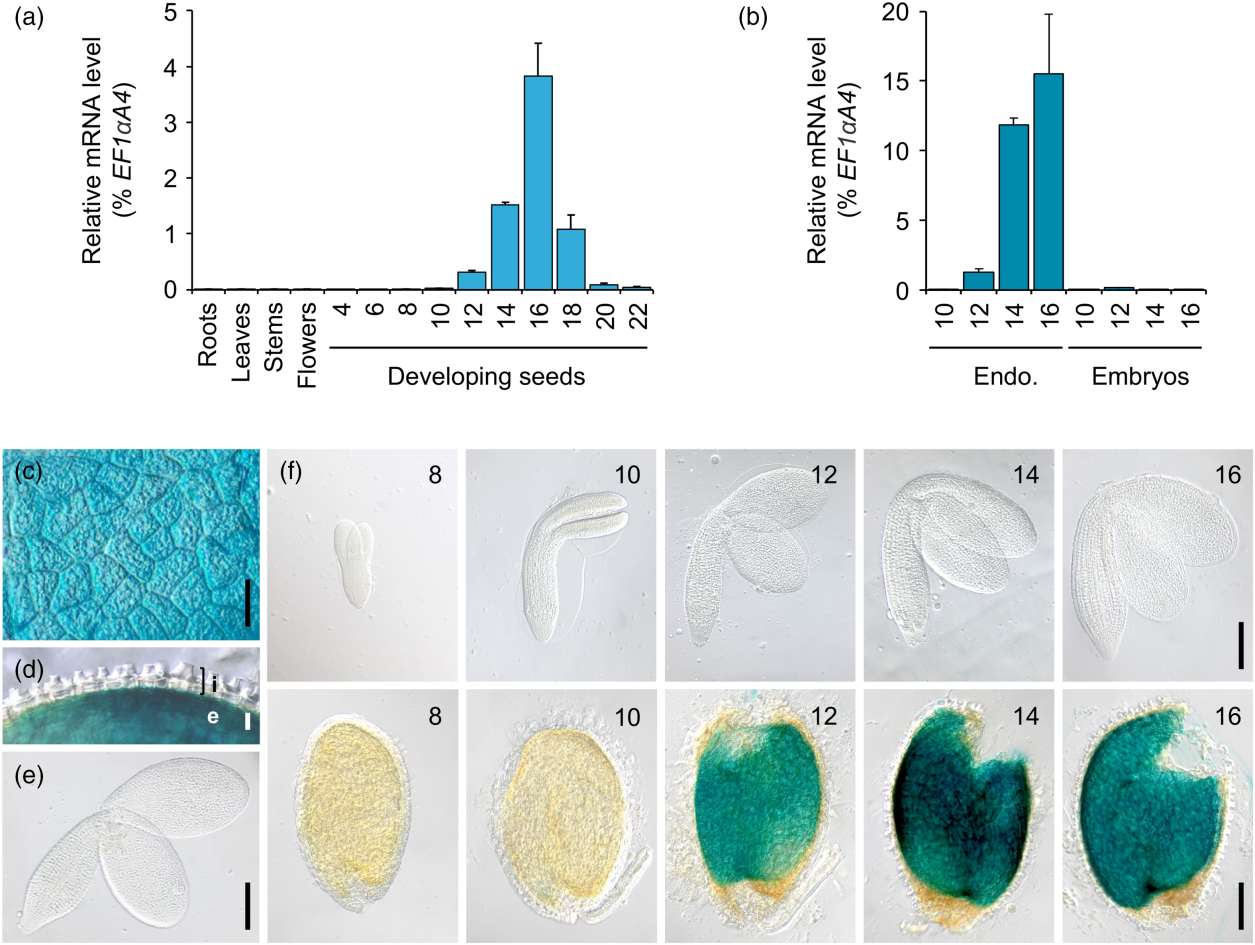
Characterization of the activity of the At3g29190 (*TPS15*) gene promoter. (a) Analysis of *TPS15* transcript accumulation was performed in different plant organs, including developing seeds harvested at different stages, from 4 to 22 days after flowering. The results obtained were standardized to the constitutive *EF1αA4* expression level. Values are means and SEs of three replicates performed on cDNA dilutions prepared from three independent mRNA extractions. (b) Analysis of *TPS15* transcript accumulation was performed in dissected seed fractions, at different stages of seed development, from 10 to 16 days after flowering. The results obtained were standardized to the constitutive *EF1αA4* expression level. Values are means and SEs of three replicates performed on cDNA dilutions prepared from three independent mRNA extractions. Endo., endosperm fraction. (c–e) Pattern of activity of the *Pro*_*TPS15*_ *:uidA* cassette in different tissues of maturing seeds. A top view of the peeled endosperm is shown in (c), a cross‐sectional view of the seed coat plus attached endosperm is shown in (d), and an embryo is shown in (e). For histochemical detection of GUS activity, dissected seed tissues were incubated overnight in a buffer containing 0.2 mM each of potassium ferrocyanide and potassium ferricyanide. Microscopic observations of seed tissues were made using Nomarski optics. e, endosperm; i, integuments. Scale bars: (c) and (d) = 20 μm, (e) = 100 μm. (f) Kinetics of the activity pattern of the *Pro*_*TPS15*_ *:uidA* cassette in maturing seeds at 8, 10, 12, 14, and 16 days after flowering. For histochemical detection of GUS activity, dissected seed tissues were incubated overnight in a buffer containing 0.2 mM each of potassium ferrocyanide and potassium ferricyanide. Microscopic observations of seed tissues were made using Nomarski optics. Scale bars = 100 μm.

In their work, Le et al. (2010) sought to identify genes that are specifically expressed in the seed and, more importantly, exclusively in certain areas of this organ. We therefore examined the list of 30 genes presented as endosperm‐specific, to which a unique AGI number had been assigned, in order to find candidates whose expression pattern in the endosperm best matched that of the players in fatty acid metabolism (Figure 1c). Of these, 24 genes are expressed at very low levels (corresponding to the blue shade 0–200) and 5 show higher expression profiles that do not coincide with the maturation phase. Only one candidate gene, At3g29190, shows an induction during the maturation phase at a level similar to that of fatty acid biosynthetic genes. The At3g29190 gene encodes an enzyme belonging to the terpene synthase (TPS) family and has been named *TPS15* (Chen et al., 2020). Phylogenetic analyses show that TPS15 belongs to the TPS‐a subfamily, which is widespread in flowering plants and comprises 22 members in *A. thaliana* (out of a total of 32 TPS) (Chen et al., 2011). Through heterologous expression in an optimized *Escherichia coli* system, TPS15 was found to have di‐terpene synthase activity (Chen et al., 2020). To confirm the transcriptomic data presented above with an independent approach, but also to better characterize the *TPS15* expression pattern throughout the seed maturation phase, we set up an RT‐qPCR approach on cDNA prepared from a range of plant organs of the wild‐type accession Columbia‐0 (Col‐0). As a first approach, a number of plant organs, including a developing series of whole seeds, were examined (Figure 2a). *TPS15* appeared to be specifically expressed in maturing seeds, with transcripts becoming detectable from 10 DAF onwards and peaking at 16 DAF. To gain further insight into the tissue specificity of *TPS15* expression, maturing seeds were dissected and the two fractions obtained, embryo and endosperm/seed coat, were analyzed independently. The time course analysis of *TPS15* mRNA abundance in these dissected seed tissues revealed a specific transcript accumulation during seed maturation in the endosperm/seed coat fraction (Figure 2b).

### Characterization of a promoter region specifically active in maturing endosperm

A 1‐kb *TPS15* promoter fragment was then cloned and transcriptionally fused to the *uidA* reporter gene, both to validate its activity and to clarify its tissue specificity. The corresponding construct was assayed for the resulting *uidA* expression pattern in transgenic *A. thaliana* lines. In the 18 independent transformants characterized in this way, the tissue specificity of the promoter activity was first considered. Intense ß‐glucuronidase (GUS) activity was observed in maturing seeds, and closer examination of dissected seed tissues revealed that while the whole endosperm showed GUS activity (Figure 2c), neither the integuments (Figure 2d) nor the embryo did (Figure 2e). Secondly, a time course analysis of the appearance of GUS activity during seed development was carried out (Figure 2f). No activity was detected at 8 DAF. Very weak activity was detected in the micropylar endosperm at 10 DAF, and it was not until 12 DAF that intense GUS activity was observed throughout the endosperm. The intensity of the staining increased until 14 DAF. Overall, these observations were very consistent with the transcript accumulation measurements previously presented. This approach confirmed that the cloned promoter was likely to be suitable for driving transgene expression during endosperm maturation in seeds of *A. thaliana*.

### Endosperm‐specific production of omega‐7 monounsaturated fatty acids

The first approach developed to validate the use of this promoter sequence consisted in the restoration of the expression of a gene that is naturally expressed in the wild‐type endosperm in the corresponding mutant background. We therefore focused on the metabolism of omega‐7 monounsaturated fatty acids, which accumulate abundantly in the endosperm of *A. thaliana* seeds in the form of palmitoleic acid (16:1^∆9^) and its elongated derivatives, vaccenic acid (18:1^∆11^) and paullinic acid (20:1^∆13^) (Penfield et al., 2004). Palmitoleic acid is mostly synthesized by the ∆9 palmitoyl‐ACP desaturases AAD2 and AAD3, and the expression of the genes encoding these desaturases is predominant in the endosperm (Troncoso‐Ponce, Barthole, et al., 2016) (Figure 1a). The enzymatic equipment needed to channel these monounsaturated fatty acids from their site of synthesis, in the stroma, to triacylglycerols, in the endoplasmic reticulum, is naturally present in *A. thaliana*.

**Figure 3:**
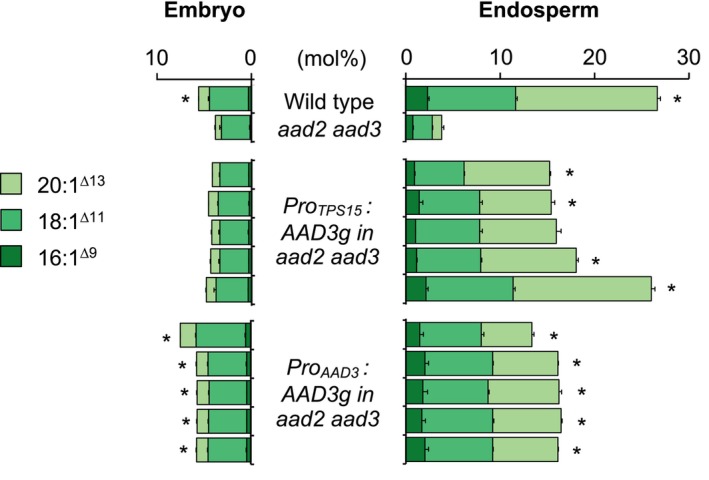
Omega‐7 fatty acid content of embryo and endosperm fraction of different transgenic lines of *Arabidopsis thaliana*. In this experiment, the *aad2 aad3* double mutant, corresponding to the genetic background in which the constructs were introduced, was used as a control. For each construct, five independent transformants were analyzed. Wild‐type plants (Col‐0 accession) were grown together with the mutants and the transformants. Each bar represents the mean and SE of five measurements made on batches of 50 organs from five different plants. Stars indicate statistically significant differences for total omega‐7 content between the respective line and the *aad2 aad3* double mutant as determined by Mann–Whitney pairwise comparisons (*P* < 0.01). A full description of the fatty acid composition can be found in Data S1.

In the endosperm fraction of *aad2 aad3* double mutant seeds, as previously reported (Bryant et al., 2016; Troncoso‐Ponce, Barthole, et al., 2016), the omega‐7 content decreased drastically compared to the wild type, from 26 to 4 mol% of total fatty acids (Figure 3). A subtle decrease was also measured in the embryo (from 6 to 4 mol%). The residual omega‐7 production measured in the *aad2 aad3* genetic background combining two knockout mutations probably reflects the low level of ∆9 palmitoyl‐ACP desaturase activity exhibited by certain ∆9 stearoyl‐ACP desaturases active in seeds, such as FAB2 (Kazaz et al., 2020), for which it has been shown that they are also able to desaturate 16:0, although this is not their preferred substrate (Kachroo et al., 2007). The genomic sequence of *AAD3*, from the ATG to the STOP codon, was cloned downstream of either the *TPS15* gene promoter or that of *AAD3*. The two constructs thus obtained were introduced into the *aad2 aad3* mutant background and, for each of them, the dissected seed fractions of five independent transformants were analyzed by gas chromatography. The use of *AAD3* promoter sequence allowed partial complementation of the mutant phenotype, with an omega‐7 content in the endosperm ranging between 13 and 16 mol%, depending on the line (Figure 3). These values, intermediate between those measured in the wild type and in the *aad2 aad3* double mutant, correspond quite well to the phenotype of the *aad2* single mutant (Troncoso‐Ponce, Barthole, et al., 2016). They confirm that when one of the two ∆9 palmitoyl‐ACP desaturases in *A. thaliana* is defective, the other is unable to fully replace it. Interestingly, using the *TPS15* gene promoter for this complementation test resulted in a better phenotypic reversion in the endosperm fraction, with the omega‐7 content varying from 15 to 26 mol% depending on the line. Taken together, these observations demonstrate that the promoter sequence tested here is perfectly capable of replacing the endogenous promoter of a gene involved in fatty acid metabolism in the endosperm of *A. thaliana* seeds.

### Endosperm‐specific production of medium‐chain fatty acids

**Figure 4:**
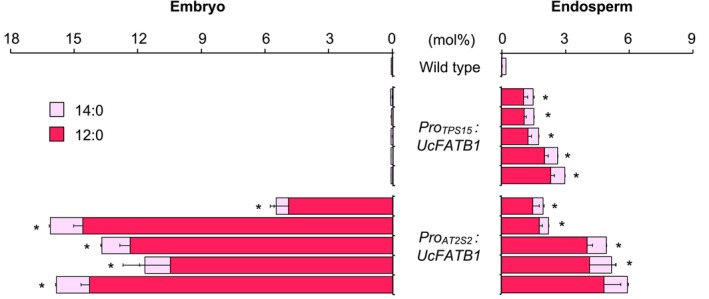
Medium‐chain fatty acid content of embryo and endosperm fraction of transgenic lines of *Arabidopsis thaliana*. In this experiment, the wild type (Col‐0 accession) was used as a control. Five independent transformants were analyzed for each construct. Each bar represents the mean and SEs of five measurements made on batches of 50 organs from five different plants. Stars indicate statistically significant differences for total medium‐chain fatty acid content between the respective line and the wild type as determined by Mann–Whitney pairwise comparisons (*P* < 0.01). A full description of the fatty acid composition can be found in Data S1.

Secondly, we wanted to test the ability of this promoter sequence to activate the expression of a gene from another species and to induce the accumulation of fatty acids normally absent in the endosperm of *A. thaliana* seeds. Medium‐chain saturated fatty acids are found only in trace amounts in wild‐type *A. thaliana* seeds (Figure 4). This is because the thioesterases present in this species only cleave acyl‐ACPs with 16 or 18 carbon atoms (Salas & Ohlrogge, 2002). In contrast, medium‐chain fatty acids are abundant in the storage lipids of certain plants, such as California bay (*U. californica*), which accumulate high levels of laurate (12:0) in its seeds. A 12:0‐ACP thioesterase, later named UcFATB1, was identified and characterized in this species, and its coding sequence was used in this experiment (Voelker et al., 1992). Five independent lines expressing *UcFAT1* under the control of the *TPS15* promoter were obtained and their seeds were analyzed (Figure 4). These seeds accumulated a significant amount of medium‐chain fatty acids in their endosperm fractions, and this accumulation was specific, as the embryo remained almost completely devoid of such fatty acids. The levels of short‐chain fatty acids measured in the endosperm ranged from 1.5 to 3 mol% of total fatty acids depending on the line. These values are in the same order of magnitude as those measured on whole seeds by Eccleston et al. (1996) when they expressed *UcFATB1* under the control of a *35S* promoter in *B. napus*. Using the promoter sequence of a napin gene, these authors then obtained seeds with higher levels of medium‐chain fatty acids and described some transgenic lines accumulating almost 60 mol% of these fatty acids (Eccleston & Ohlrogge, 1998; Voelker et al., 1996). However, it appears that a large number of lines had to be screened before such results could be obtained, as most of these lines tended to have levels of around 10 mol% of medium‐chain fatty acids. We also ignore whether the fatty acid composition of the endosperm is similar to that measured at the whole seed level in these high laurate lines. To further investigate this aspect, we expressed *UcFATB1* under the control of the *A. thaliana AT2S2* promoter: *AT2S2* encodes a napin and is highly expressed in both embryo and endosperm (Ettaki et al., 2018). In the *Pro*_*AT2S2*_ *:UcFATB1* lines characterized, the content of medium‐chain fatty acids in the embryo varied between 5 and 15 mol% (Figure 4), a range of values quite comparable with the average results obtained on whole seeds in *B. napus* using a napin promoter to drive the expression of *UcFATB1* (Eccleston & Ohlrogge, 1998). Strikingly, in each of the *Pro*_*AT2S2*_ *:UcFATB1* lines obtained, the levels of medium‐chain fatty acids in the endosperm were lower than those measured in the embryo (by three‐ to sevenfold). As a consequence, the levels of medium‐chain fatty acids in the endosperm of the *Pro*_*AT2S2*_ *:UcFATB1* lines were only slightly higher than those measured in the *Pro*_*TPS15*_ *:UcFATB1* lines. While the transcriptomic (Le et al., 2010) and RT‐qPCR data (Ettaki et al., 2018) suggest that *AT2S2* promoter activity is comparable in the two zygotic tissues of maturing seeds, the results obtained here suggest that specific characteristics of lipid metabolism in the endosperm make it less able to take up medium‐chain fatty acids and channel them into triacylglycerols.

Taken together, these results show that the *TPS15* gene promoter is capable of inducing endosperm‐specific accumulation of fatty acids normally absent from this compartment in *A. thaliana* seeds. In the case of medium‐chain fatty acids, this specificity of action is slightly offset by a relative loss of efficacy compared to the use of the non‐specific *AT2S2* promoter. It would now be interesting to co‐express *UcFATB1* in the endosperm with one or more sequences encoding acyltransferases specialized in the acylation of medium‐chain fatty acids, such as *EgDAGT1‐1* from *E. guineensis* (Aymé et al., 2015; Dussert et al., 2013), in order to test whether it is possible to increase the concentration of medium‐chain fatty acids in this tissue.

### Endosperm‐specific repression of polyunsaturated fatty acid biosynthesis

**Figure 5:**
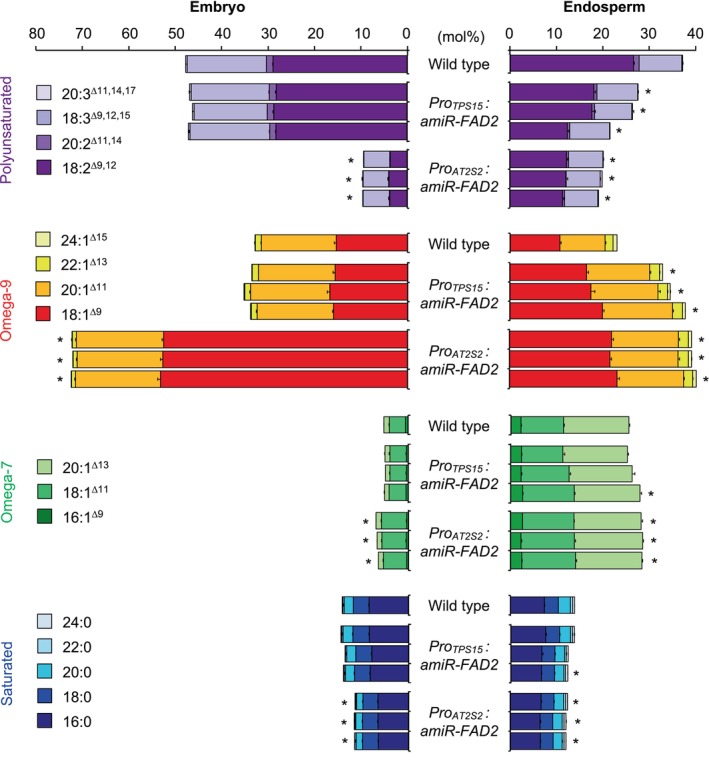
Fatty acid composition of embryo and endosperm fraction of transgenic lines of *Arabidopsis thaliana* with repressed *FAD2* expression. In this experiment, the wild type (Col‐0 accession) was used as a control. Three independent transformants were analyzed for each construct. Each bar represents the mean and SEs of five measurements made on batches of 50 organs from five different plants. Stars indicate statistically significant differences for a given fatty acid category (polyunsaturated, omega‐9, omega‐7, or saturated) between the respective line and the wild type as determined by Mann–Whitney pairwise comparisons (*P* < 0.01). A full description of the fatty acid composition can be found in Data S1.

While the two previous examples involved the endosperm‐specific induction of a gene no longer expressed or not expressed in this seed compartment, we then wanted to test whether it was possible to specifically repress the expression of a gene in the endosperm using the *TPS15* promoter sequence. To this end, we chose to target the gene encoding the ∆12 desaturase FAD2 in order to reduce the level of polyunsaturated fatty acids in the endosperm, which make up 37 mol% of total fatty acids in the wild type (Figure 5). A modified version of the *A. thaliana miR159b* gene containing a 21mer sequence targeting *FAD2* without affecting off‐target genes was used (Belide et al., 2012). This cassette was placed under the control of the *TPS15* gene promoter. As a control, the same cassette was cloned downstream of the *AT2S2* promoter. In transgenic lines carrying the *Pro*_*AT2S2*_ *:amiR‐FAD2* construct, a significant reduction of polyunsaturated fatty acid content was observed both in the embryo (about 80% compared to the wild type) and in the endosperm (about 50% compared to the wild type) (Figure 5). This loss of polyunsaturated fatty acids was essentially compensated by an overaccumulation of oleate (18:1^∆9^). Once again, there was a difference in the efficiency of the construct between the two zygotic tissues, to the disadvantage of the endosperm, which is difficult to explain given the current state of knowledge of lipid metabolism in the endosperm. It is interesting to compare these observations with those made by Sturtevant et al. (2017) when they analyzed the distribution of membrane phospholipids in *A. thaliana* seeds using MALDI‐MS. When characterizing the *fad2‐1* mutant seeds, the authors found that the concentration of certain phospholipids carrying polyunsaturated fatty acids, such as PC 34:3, was less affected by the *fad2‐1* mutation in the endosperm than in the embryonic tissues. This observation led the authors to postulate alternative means of desaturating 18:1^∆9^ to 18:2^∆9,12^ in the endosperm.

We then characterized lines expressing the *Pro*_*TPS15*_ *:amiR‐FAD2* construct and observed a specific decrease in the polyunsaturated fatty acid content in the endosperm. The level of polyunsaturated fatty acids in the endosperm of the transgenic lines was reduced by 25–40% compared to the level measured in the wild type. Once again, it seems that the specificity of the *TPS15* promoter is offset by a slight loss of efficiency compared to the use of the napin promoter.

## CONCLUSION

In conclusion, we have shown that the *TPS15* promoter sequence studied, which is specifically active in the endosperm during seed maturation, can be used to effectively modulate the fatty acid composition of seed endosperm without altering that of the embryo, using overexpression or silencing approaches. It is interesting to note that of the few genes whose expression is specific to the endosperm of seeds according to Le et al. (2010), *TPS15* seems to be the only one whose activation coincides temporally with the seed maturation phase. This could be explained by the fact that the seed maturation process and associated genes are regulated by master regulators that are expressed in both zygotic tissues of the *A. thaliana* seed, so that even if aspects of maturation often differ between the two seed compartments, they are usually variations on the same theme (Barthole et al., 2014). Nevertheless, it is not impossible that, with the advent of single‐cell sequencing techniques (Liew et al., 2024), it will soon be possible to obtain a new and finer vision of the transcriptome in the different territories of the developing seed, and thus to reveal new candidates specifically expressed in the endosperm during maturation. Meanwhile, it would be interesting to carry out a detailed functional characterization of the *TPS15* promoter sequence in order to try to understand the molecular determinisms of its unique activity. This knowledge could lead us to be more effective in biotechnological approaches aimed at modifying endosperm metabolism, possibly by creating a synthetic promoter that is as specific as the native *TPS15* promoter and even more active during the maturation phase.

These approaches could prove invaluable for bioengineering the oil composition of seeds, since, as this study has shown, the promoters of reserve protein genes used to date appear to be much less effective in the endosperm than in the embryo. However, this obviously raises the question of the conservation between species of the molecular mechanisms underlying the activity of the *TPS15* promoter, an important issue to be studied in different oilseed species, starting with Brassicaceae oilseeds related to the model species *A. thaliana*, before even considering important oleaginous species with developed endosperm tissues, such as castor bean or oil palm.

Finally, the tools tested here and those to be developed in the future will allow us to better characterize the lipid metabolism of the endosperm, which, according to the data collected here and available in the literature, differs in many respects from that of the embryo. This may also help us to understand the physiological significance of the specificities of lipid metabolism in the endosperm. For example, polyunsaturated fatty acids are particularly susceptible to oxidation, and since fatty acid peroxidation is an autocatalytic process, their oxidation can be devastating if uncontrolled (Zinsmeister et al., 2020). This process is considered to be a predominant damaging factor during oilseed aging (Fabrissin et al., 2021). It would be interesting to test whether limiting the proportion of polyunsaturated fatty acids in the endosperm, a peripheral tissue of the seed that is more exposed to free radicals, can reduce the loss of seed viability during aging. Another unexplained observation linking fatty acid metabolism to seed physiology concerns the negative effect of high levels of saturated fatty acids (e.g., stearate) on seed germination at low temperatures (Kazaz et al., 2020). It remains unclear at this stage what mechanism prevents germination in lines rich in saturated fatty acids, and whether this block originates from the endosperm, the embryo, or both tissues. However, the biological questions related to the specificities of the endosperm compared to the embryo are numerous and certainly not limited to the field of lipid metabolism. For example, this promoter may also prove to be a valuable tool for studying the molecular dialogs that occur between the endosperm and the embryo during seed development.

## EXPERIMENTAL PROCEDURES

### Plant material and growth conditions

*Arabidopsis thaliana* seeds of the Col‐0 (Columbia‐0) accession were obtained from the Plant Observatory—*A. thaliana* Stock Centre at the Institute Jean‐Pierre Bourgin for Plant Sciences. T‐DNA mutant lines (*aad2‐3*, N670942; *aad3‐3*, N567280) were ordered from the Eurasian Arabidopsis Stock Centre and were previously described in Troncoso‐Ponce, Barthole, et al. (2016). Seeds were sterilized and germinated as described in Baud et al. (2007). Briefly, after a cold treatment of 48 h at 4°C in the dark, the plates were kept in a growth chamber (16‐h light/21°C, 8‐h dark/19°C, 200 μE m^−2^ sec^−1^, 65% relative humidity). After 10 days, the plantlets were transferred to soil (Tref substrates), grown in a greenhouse with a minimum photoperiod of 13 h ensured by supplementary lighting and irrigated with Plan‐Prod nutrient solution (Fertil). In each experiment, all the genotypes studied were grown together. To sample embryo and endosperm fractions, seeds excised from siliques were dissected using a scalpel and dissecting tweezers under a binocular optical glass loupe. The material was frozen in liquid nitrogen immediately after harvest and then stored at −80°C. The two fractions obtained, embryos on one side and endosperm with seed coat on the other, were analyzed separately. Considering that the seed coat undergoes programmed cell death during the maturation phase (Beeckman et al., 2000), the analysis of the second fraction reflected the lipid content of the endosperm alone.

### Constructs

The sequences of primers used for DNA amplification are indicated in Table S1.

To prepare the *Pro*_*TPS15*_ *:uidA* construct, a DNA fragment containing a 1‐kb *TPS15* promoter sequence was amplified from Col‐0 genomic DNA using the proofreading DNA polymerase Pfu Ultra (Stratagene). The PCR product was inserted into the pDONR207 entry vector (Invitrogen) by BP recombination and transferred into the pGWB3 destination vector (Nakagawa et al., 2007) by LR recombination. The resulting binary vector was electroporated into *Agrobacterium tumefaciens* strain C58C^1^ and used for agroinfiltration of *A. thaliana* flower buds (Bechtold et al., 1993). Primary transformants were selected on MS medium containing hygromycin (50 mg L^−1^) and transferred to soil for further characterization: 18 independent transgenic lines were analyzed.

To prepare the *Pro*_*AAD3*_ *:AAD3g* construct, a DNA fragment containing a 2022‐bp *AAD3* promoter sequence plus open reading frame was amplified from Col‐0 genomic DNA using the proofreading DNA polymerase Pfu Ultra. The PCR product was inserted into the pDONR207 entry vector by BP recombination and transferred into the pBIB‐Hyg‐GTW destination vector (Dubos et al., 2008) by LR recombination. After agroinfiltration, primary transformants were selected on MS medium containing hygromycin.

To prepare the *Pro*_*TPS15*_ *:AAD3g* construct, a DNA fragment containing a 1‐kb *TPS15* promoter sequence and a DNA fragment containing the *AAD3* open reading frame were amplified from Col‐0 genomic DNA using the proofreading DNA polymerase Pfu Ultra. After purification using the Gel and PCR Clean‐up kit (Macherey‐Nagel), the two DNA fragments, which have a complementary sequence at one of their ends due to the design of the primers chosen for their amplification, were diluted 1:10, mixed and used as a template for amplification of the *Pro*_*TPS15*_ *:AAD3g* fragment using the DNA polymerase Pfu Ultra. The PCR product was inserted into the pDONR207 entry vector by BP recombination and transferred into the pBIB‐Hyg‐GTW destination vector by LR recombination. After agroinfiltration, primary transformants were selected on MS medium containing hygromycin.

To prepare the *Pro*_*AT2S2*_ *:UcFATB1* construct, a DNA fragment containing the coding sequence of *UcFATB1* was amplified from a DNA matrix synthesized (Twist Bioscience) according to GenBank: M94159.1 using the proofreading DNA polymerase Pfu Ultra. The PCR product was inserted into the pDONR207 entry vector by BP recombination and transferred into the Pro_AT2S2_‐R1R2‐HYGRO destination vector (Pouvreau et al., 2011) by LR recombination. After agroinfiltration, primary transformants were selected on MS medium containing hygromycin.

To prepare the *Pro*_*TPS15*_ *:UcFATB1* construct, a DNA fragment containing a 1‐kb *TPS15* promoter sequence was amplified from Col‐0 genomic DNA and a DNA fragment containing the *UcFATB1* coding sequence was amplified from the DNA matrix synthesized (Twist Bioscience) according to GenBank: M94159.1. After purification, the two DNA fragments, which have a complementary sequence at one of their ends due to the design of the primers chosen for their amplification, were diluted 1:10, mixed and used as a template for amplification of the *Pro*_*TPS15*_ *:UcFATB1* fragment using the DNA polymerase Pfu Ultra. The PCR product was inserted into the pDONR207 entry vector by BP recombination and transferred into the pBIB‐Hyg‐GTW destination vector by LR recombination. After agroinfiltration, primary transformants were selected on MS medium containing hygromycin.

To prepare the *Pro*_*AT2S2*_ *:amiR‐FAD2* construct, a DNA fragment containing the *amiR‐FAD2* cassette was amplified from a DNA matrix synthesized (Twist Bioscience) according to Belide et al. (2012). The PCR product was inserted into the pDONR207 entry vector by BP recombination and transferred into the Pro_AT2S2_‐R1R2‐HYGRO destination vector by LR recombination. After agroinfiltration, primary transformants were selected on MS medium containing hygromycin.

To prepare the *Pro*_*TPS15*_ *:amiR‐FAD2* construct, a DNA fragment containing a 1‐kb *TPS15* promoter sequence was amplified from Col‐0 genomic DNA and a DNA fragment containing the *amiR‐FAD2* cassette was amplified from the DNA matrix synthesized (Twist Bioscience) according to Belide et al. (2012). After purification, the two DNA fragments, which have a complementary sequence at one of their ends due to the design of the primers chosen for their amplification, were diluted 1:10, mixed and used as a template for amplification of the *Pro*_*TPS15*_ *:amiR‐FAD2* fragment using the DNA polymerase Pfu Ultra. The PCR product was inserted into the pDONR207 entry vector by BP recombination and transferred into the pBIB‐Hyg‐GTW destination vector by LR recombination. After agroinfiltration, primary transformants were selected on MS medium containing hygromycin.

The progeny of the primary transformants (T2 seeds) was subjected to segregation analyses, and lines segregating 3:1 for hygromycin resistance were selected. T2 lines were then propagated, and their progeny (T3 seeds) was subjected to segregation analyses as well. Homozygous lines producing 100% resistant plantlets were selected and used for biochemical analysis.

### Fatty acid analyses

Total fatty acid methyl esters were prepared from 50 seed fractions as previously described (Li et al., 2006). Briefly, samples were incubated in a glass reaction tube in 1.3 mL of methanol/sulfuric acid/toluene (100:2.5:30, v/v/v) at 94°C for 90 min. Fatty acyl methyl esters were then extracted in 0.5 mL of hexane after the addition of 1.5 mL of a 154 mM NaCl solution. After vigorous shaking and centrifugation, 1 μL of the upper organic phase was analyzed by gas chromatography (Agilent, 7890B GC system) on a CP‐Wax 52 CB 30 m × 530 μm × 1 μm column (Agilent). Fatty acid methyl esters were identified by their retention time relative to standards and quantified using 17:0 for calibration. The gas chromatographic acquisition parameters were as follows: hydrogen was used as carrier gas at the rate of 5.25 mL min^−1^; injector temperature of 250°C; detector temperature of 250°C; oven initial temperature of 175°C for 0 min, followed by a ramp of 0.5°C min^−1^ to 190°C, then a ramp of 3°C min^−1^ to 244°C, this final temperature being held for 5 min; splitless injection.

### RNA analyses

RNA extraction and reverse transcription (RT) were performed as previously described (Baud et al., 2004). Real‐time quantitative PCR reactions were performed in a CFX Connect Real‐Time PCR Detection System (Biorad), using the SsoAdvanced PreAmp Supermix Biorad kit according to the manufacturer's protocol. Reactions used 5 μL of 1:50 diluted cDNA in a total volume of 15 μL. The reactions were incubated as follows: a first step at 95°C for 8 min to activate the hot start recombinant Taq DNA polymerase, followed by 40 cycles of 95°C for 10 sec and 60°C for 10 sec. The specificity of the PCR amplification was checked with a heat dissociation protocol (from 60 to 95°C with a temperature transition rate of 0.1°C sec^−1^) after the last cycle of the PCR. The results obtained were standardized to the level of expression of the constitutive *EF1αA4* gene (encoding a translation elongation factor) (Nesi et al., 2000). The sequences of the primers used for real‐time PCR are given in Table S2. The efficiency of the primer sets was calculated by performing real‐time PCR on several dilutions of the first strands. The efficiencies of the different primer sets used were verified to be close to each other.

### Microscopy

Histochemical detection of GUS activity and bright‐field microscope observations were performed as described in Baud et al. (2007).

## ACCESSIONS NUMBERS

Arabidopsis sequence data from the present study are available in the EMBL/GenBank data libraries under accession numbers: *AAD2*, At3g02610; *AAD3*, At5g16230; *EF1αA4*, At5g60390; *FAD2*, At3g12120; *TPS15*, At3g29190.

## AUTHOR CONTRIBUTIONS

SB designed the experiments. AT, SK, RM, and SB performed the experiments. RM and SB analyzed the data. SB conceived and wrote the paper, which was revised and approved by all authors.

## CONFLICT OF INTEREST

The authors declare no conflict of interest.

## Supporting information


**Data S1.** Detailed fatty acid composition of seed fractions from the transgenic lines characterized in this study.

**Table S1.** Primers used for construct preparation.
**Table S2.** Primers used for real time PCR.

## Data Availability

The data that support the findings of this study are available on request from the corresponding author. The data are not publicly available due to privacy or ethical restrictions.
