# Supplementary material for: Identification of a promoter region specifically active in the maturing endosperm of Arabidopsis seeds and its use for targeted modification of fatty acid metabolism
Source: Plant J. 2025 Mar 3;121(5):e70038. doi: 10.1111/tpj.70038 (PMC11874189; doi:10.1111/tpj.70038)
Supplement: Supplementary file 2 — Table S1. Primers used for construct preparation. Table S2. Primers used for real time PCR. [file TPJ-121-0-s002.pdf]

Table S1. Primers used for construct preparation

Construct	Sequence cloned	Primer	Primer sequence (5'→3')
Pro$_{TPS15}$*:uidA*	*At3g29190* (1-kb promoter sequence)	Fw	GGGGACAAGTTTGTACAAAAAAGCAGGCTTCGGAGATGTATTTTTTTGTGTG
		Rv	GGGGACCACTTTGTACAAGAAAGCTGGGTCGTTCACTACAAATGATATAGAATG
Pro$_{AAD3}$*:AAD3g*	*At5g16230* (2-kb promoter sequence + ORF)	Fw	GGGGACAAGTTTGTACAAAAAAGCAGGCTTCATGCCGTTTGCTGATATG
		Rv	GGGGACCACTTTGTACAAGAAAGCTGGGTCATCTATCTATAGTTTCACATCTCTACC
Pro$_{TPS15}$*:AAD3g*	*At3g29190* (1-kb promoter sequence)	Fw	GGGGACAAGTTTGTACAAAAAAGCAGGCTTCGGAGATGTATTTTTTTGTGTG
		Rv	CAAAGCCATCGACATGTTCACTACAAATGATATAGAATG
Pro$_{TPS15}$*:AAD3g*	*At5g16230* (ORF)	Fw	CATTCTATATCATTTGTAGTGAACATGTCGATGGCTTTG
		Rv	GGGGACCACTTTGTACAAGAAAGCTGGGTCATCTATCTATAGTTTCACATCTCTACC
Pro$_{AT2S2}$*:UcFATB1*	*UcFATB1* (CDS)	Fw	GGGGACAAGTTTGTACAAAAAAGCAGGCTTCATGGCCACCACCTCT
		Rv	GGGGACCACTTTGTACAAGAAAGCTGGGTCTACACCCTCGGTTCTG
Pro$_{TPS15}$*:UcFATB1*	*At3g29190* (1-kb promoter sequence)	Fw	GGGGACAAGTTTGTACAAAAAAGCAGGCTTCGGAGATGTATTTTTTTGTGTG
		Rv	AGAGGTGGTGGCCATGTTCACTACAAATGATATAGAATG
Pro$_{TPS15}$*:UcFATB1*	*UcFATB1* (CDS)	Fw	CATTCTATATCATTTGTAGTGAACATGGCCACCACCTCT
		Rv	GGGGACCACTTTGTACAAGAAAGCTGGGTCTACACCCTCGGTTCTG
Pro$_{AT2S2}$*:amiR-FAD2*	*amiR-FAD2* cassette	Fw	GGGGACAAGTTTGTACAAAAAAGCAGGCTTCACTAGTGATTTCACTTTTGTTCTC
		Rv	GGGGACCACTTTGTACAAGAAAGCTGGGTCTTCGAACCCAGACACTTA
Pro$_{TPS15}$*:amiR-FAD2*	*At3g29190* (1-kb promoter sequence)	Fw	GGGGACAAGTTTGTACAAAAAAGCAGGCTTCGGAGATGTATTTTTTTGTGTG
		Rv	GAGAACAAAAGTGAAATCACTAGTGTTCACTACAAATGATATAGAATG
Pro$_{TPS15}$*:amiR-FAD2*	*amiR-FAD2* cassette	Fw	CATTCTATATCATTTGTAGTGAACACTAGTGATTTCACTTTTGTTCTC
		Rv	GGGGACCACTTTGTACAAGAAAGCTGGGTCTCGAACCCAGACACTTA

Sequences in blue denote DNA recombination sequences (att sites). The sequences shown in green are extensions added to the 5' of certain primers to allow hybridization of two different PCR products.

CDS, coding sequence; Fw, forward primer; ORF, open reading frame; Rv, reverse primer.

Table S2. Primers used for real time PCR

Gene	AGI	Forward primer (5'→3')	Reverse primer (5'→3')
TPS15	At3g29190	AGAGGATATGCGGCGAATGC	ATGAGTCCAACGCCCATTGC
EF1αA4	At5g60390	CTGGAGGTTTTGAGGCTGGTAT	CCAAGGGTGAAAGCAAGAAGA
